# Multisite Attenuated Intracellular Recordings by Extracellular Multielectrode Arrays, a Perspective

**DOI:** 10.3389/fnins.2018.00212

**Published:** 2018-04-10

**Authors:** Micha E. Spira, Nava Shmoel, Shun-Ho M. Huang, Hadas Erez

**Affiliations:** Department of Neurobiology, The Alexander Silberman Institute of Life Science, The Charles E Smith Family and Prof. Joel Elkes Laboratory for Collaborative Research in Psychobiology, The Harvey M. Kruger Family Center for Nanoscience, Hebrew University of Jerusalem, Jerusalem, Israel

**Keywords:** electrophysiology, multielectrode-array, action-potential, synaptic-potentials, hippocampus, cardiomyocytes, Aplysia, skeletal- myotubes

## Abstract

Multielectrode arrays (MEA) are used extensively for basic and applied electrophysiological research of neuronal- and cardiomyocyte-networks. Whereas immense progress has been made in realizing sophisticated MEA platforms of thousands of addressable, high-density, small diameter, low impedance sensors, the quality of the interfaces formed between excitable cells and classical planar sensor has not improved. As a consequence *in vitro* and *in vivo* MEA are “blind” to the rich and important “landscape” of sub-threshold synaptic potentials generated by individual neurons. Disregarding this essential fraction of network signaling repertoire has become the standard and almost the “scientific ideology” of MEA users. To overcome the inherent limitations of substrate integrated planar MEA platforms that only record extracellular field potentials, a number of laboratories have developed *in vitro* MEA for intracellular recordings. Most of these novel devices use vertical nano-rods or nano-wires that penetrate the plasma membrane of cultured cells and record the electrophysiological signaling in a manner similar to sharp intracellular microelectrodes. In parallel, our laboratory began to develop a bioinspired approach in-which cell biological energy resources are harnessed to self-force a cell into intimate contact with extracellular gold mushroom-shaped microelectrodes to record attenuated synaptic- and action-potentials with characteristic features of intracellular recordings. Here we describe some of the experiments that helped evolve the approach and elaborate on the biophysical principles that make it possible to record intracellular potentials by an array of extracellular electrode. We illustrate the qualities and limitations of the method and discuss prospects for further improvement of this technology.

## Introduction

Multi-electrode arrays (MEA) are extensively used for basic and applied electrophysiological research of *in vivo* and *in vitro* neuronal and cardiomyocyte networks (Obien et al., 2014; Seymour et al., 2017). The core technology and concepts of contemporary MEA goes back half a century to the pioneering studies of Wise et al. (1970, *in vivo*) and Thomas et al. (1972, *in vitro*).

Whereas immense progress has been made over the last 50 years in realizing sophisticated *in vitro* and *in vivo* MEA platforms of thousands of addressable, high-density, small diameter low impedance sensors (for example, Berdondini et al., 2005, 2009; Amin et al., 2016; Jäckel et al., 2017; Jun et al., 2017; Viswam et al., 2017), the quality of the interfaces formed between cultured neurons or cardiomyocytes and the substrate integrated planar sensor has not improved in any significant manner. In fact, this interface remains the weakest link in the chain of electrical coupling between cultured cells and planar electrodes. As in the past, current day *in vitro* and *in vivo* planar sensor based MEAs are limited to recordings of extracellular field potentials (FPs) generated by propagating action potentials (APs). These electrodes are “blind” to the rich and important “landscape” of sub-threshold inhibitory, excitatory and electrotonic synaptic potentials generated by individual neurons. As a result, neurons that do not fire APs are not noted. Since in some brain areas and possibly also in cultured neuronal networks, a fraction of the cells fire at very low rates or do not fire at all, their potential contribution to information processing and integration go undetected and ignored (Shoham et al., 2006; Epsztein et al., 2011; Barth and Poulet, 2012). This disregarding of an essential fraction of the signaling repertoire in information processing and plasticity has become the standard and practically speaking the “scientific ideology” for many MEA users. This is the case despite unequivocal documentation that meaningful subthreshold signaling between neurons plays critical roles in neuronal network computations (Spira and Hai, 2013). This irrational neglect and standard reflect a failure of the neuroscientific community to communicate the need to develop novel electrophysiological technologies that will enable to record the entire brain-signaling repertoire. The outstanding successes in harnessing chronic *in vivo* FP recordings to functionally interface CNS neuronal activity and prostatic devices, to link disrupted groups of neurons, operate cochlear and retinal implants validate the immense importance and contribution of extracellular MEA to basic and applied brain research. However, extracellular recordings of FP-spatiotemporal patterns are not sufficient in themselves to understand how neuronal networks composed of excitatory and inhibitory neurons with heterogeneous membrane properties and synapses compute and undergo activity dependent plastic changes. Likewise, the popular use of planar MEA to extract the mechanisms of information processing, or screen toxins and drugs by recording FPs do not provide sufficient information vis a vis the mechanism by which a network operate and drugs exert their effects. Recall that alterations in membrane excitability or changes in excitatory or inhibitory synaptic efficacies may produce undistinguishable changes in the firing patterns of excitable cells networks.

Cognizant of the biophysical source for the inherent limitations of substrate integrated planar MEA platforms to only record extracellular FPs and the immense contribution expected from the development of multisite long-term “intracellular capable” MEA-platforms to brain research, a small number of laboratories have elegantly begun to develop *in vitro* MEA for intracellular recordings (for a review (Spira and Hai, 2013; Angle et al., 2015). The majority of these devices use passive or active (transistorized) nano-rods or nano-wires that are designed to penetrate the plasma membrane of cultured cells and record the electrophysiological signaling in a way similar to classical sharp intracellular microelectrodes. Because the diameter of the nano-rods (wires) is in the range of 50–500 nm, the penetration of the sensors through the plasma membrane does not damage the cells. This approach has been successfully used to record both attenuated APs and synaptic potentials from neurons and cardiomyocytes (Tian et al., 2010; Angle and Schaefer, 2012; Duan et al., 2012; Gao et al., 2012; Robinson et al., 2012; Xie et al., 2012; Angle et al., 2014; Lin and Cui, 2014; Lin et al., 2014; Qing et al., 2014; Dipalo et al., 2017; Liu et al., 2017). Recently the laboratory of H. Park (Abbott et al., 2017) reported the simultaneous recordings of attenuated APs from hundreds of cultured primary cardiomyocytes.

Along with the exciting development of cell-penetrating nano-structures technologies, our laboratory began to test a different approach in which micrometer-sized, extracellular gold mushroom-shaped microelectrodes (gMμEs) record attenuated synaptic and APs with characteristic features of intracellular recordings (Spira et al., 2007; Hai et al., 2010a,b; Fendyur and Spira, 2012; Spira and Hai, 2013; Rabieh et al., 2016; Shmoel et al., 2016). In this manuscript we first describe some of the experiments that helped develop this approach and clarified the biophysical principles that enable to record attenuated intracellular potentials by an array of extracellular electrodes. Next we briefly illustrate the qualities and limitations of the intracellular recordings obtained by the extracellular gMμE-MEA, and finally briefly discuss prospects for further improvement of this technology.

## The biophysical principles that enable cell-noninvasive extracellular electrodes to record intracellular potentials

**Figure 1:**
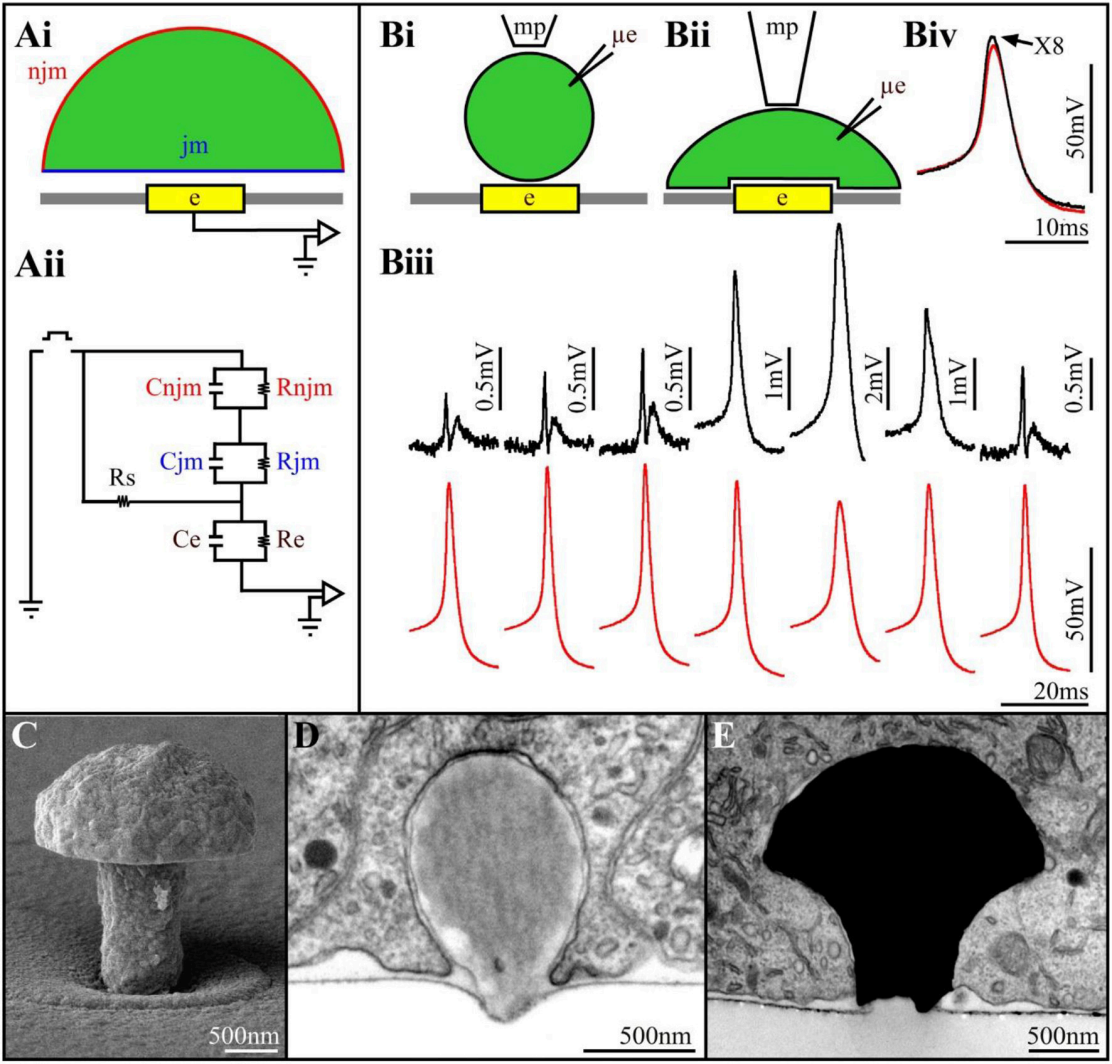
**(Ai)** Schematic drawing of a cell (green) residing on a planar sensing electrode (yellow) and the space between them (white). **(Aii)** An analog electrical circuit of the cell-electrode interface. In the model, the cell's surface area is subdivided into a non-junctional membrane (njm, red) that faces the grounded culture medium, and a junctional membrane (jm, blue) that faces the electrode. Each of these membrane compartments is represented by a resistor and capacitor in parallel R_njm_, C_njm_, R_jm_, and C_jm_, respectively. The cleft between the cell and the sensor is represented by a resistor (the seal resistance-Rs). The electrode is represented by a resistor and capacitor (Re, Ce, respectively). The electrical coupling coefficient (CC) between a cell and a recording device is defined as the ratio between the voltage recorded by the device (electrode-amplifier) and the voltage generated across the plasma membrane of an excitable cell (V_elect_/V_cell_). The square pulse in between the ground jm and njm is a voltage calibration pulse **(Bi,Bii)** schematic illustrations of downward displacement of a cell to increase the seal resistance (mp-a fire polished pipette to exert the pressure, μe-intracellular recording electrode). **(Biii)** Concomitant alterations in the extracellularly recorded FPs (upper traces- black) and intracellular APs (lower traces- red) during the displacement of the neuron's cell body toward the planar electrode. From left to right, initially the increased FPs amplitude is not associated with changes in the intracellular AP amplitude. Thereafter (4th trace), the extracellular FP recorded by the planar electrode transformed to intracellular recordings of an AP. This is associated by reduction in the AP amplitude recorded by the μE. Release of the mechanical pressure led to the reversal of the process (traces 6 and 7). **(Biv)** Super positioning of the intracellular recorded APs with the sharp electrode and the extracellular planar electrode (multiplied by 8). Note that although the amplitudes of the two APs are different the shapes of the APs are similar (**Biii,Biv** modified with permission from Cohen et al., 2008). **(C)** A scanning electron microscope image of a gMμE. **(D)** a latex bead engulfed by a cultured Aplysia neuron. **(E)** An electron microscope image of a thin section showing a gMμE (black) tightly engulfed by a cultured PC12 cell.

The mode and quality of the electrical coupling between an excitable cell and a substrate integrated planar electrode are defined by the spatial relationships and electrical properties of the living cells, the sensing device and the space between the two (Figure 1A). A simplified analog electrical circuit of a cultured neuron adhering to a substrate integrated planar electrode is depicted in Figure 1A.

Changing the quantitative relationships between the seal resistance, junctional membrane properties and the electrode impedance will therefore change the mode (extracellular to intracellular), quality (time derivative of the AP to Ohmic recordings) and amplitude (from microvolts to tens of mV) of the recorded potentials (Fromherz, 2003; Shmoel et al., 2016). Along with advances in material science, significant progress has been made over the years in the fabrication of low impedance planar electrodes. Despite extensive attempts to increase the seal resistance increasing the adhesion of the cells to the substrate in practice, the seal resistance has remained relatively low, and sufficient to shunt a large fraction of the currents generated by propagating APs to the bath ground. To the best of our knowledge, no studies have reported successful attempts to locally increase the conductance of the membrane patch facing substrate integrated planar electrodes for long durations.

Two studies have however demonstrated the exceptional potential to generate effective electrical coupling between cells and integrated planar electrodes. That of Jenkner and Fromherz (1997) using isolated leech neurons followed by that of Cohen et al. (2008) using *Aplysia* neurons. Both studies demonstrated that when a neuron's cell body, or an axon, is mechanically displaced downwards toward the surface of a planar sensor, the contacting surface area between the cell and the substrate increases gradually (Figure 1B). This gradual increase is accompanied by increased FP amplitude (recorded by the planar electrode) but without a significant change in its shape (Figure 1B). Concomitant intracellular recordings of the APs by a sharp glass electrode revealed that the intracellular spike amplitude and shape were not altered. This increased amplitude of the extracellular FP was attributed to increased R_seal_ due to the increased contact area between the neuron and the sensor, and possibly also to reduction in the average cleft width (Figure 1B). Surprisingly, further increases in the mechanical pressure transformed the recorded extracellular FP (by the planar sensor) into positive monophasic attenuated APs with characteristic features of classical intracellular recordings (Figure 1B). The transformation of an extracellular FP into an intracellular attenuated AP was associated with decreased amplitude of the intracellularly recorded AP by the sharp glass electrode. This indicated that stretching the neuron's plasma membrane against the substrate led in addition to the increased R_seal_ to a transformation of the coupling from capacitive to Ohmic apparently due to the increase in the conductance of the membrane facing the planar electrode. Releasing the mechanical pressure led to the reversal of all the parameters, including the amplitude of the intracellularly recorded AP.

## Cell biological energy resources to intimately interface cells and microelectrodes

The use of external force to mechanically manipulate the tip of an electrode into contact with a cell (as is done in classical sharp or patch electrodes) or to manipulate a cell into contact with a flat electrode (as described above) cannot be adopted across the board for multisite, long-term, electrophysiological recordings. We hypothesized that an alternative way to achieve this goal would be to engage cell biological energy resources to self-force a cell into intimate contact with an electrode. To that end our laboratory began to examine this possibility by deviating from the “traditional” planar MEA fabrication technology to the use of 3D electrode structures that mimic neuronal structures in terms of their shape and dimensions. Our initial hypothesis (Spira et al., 2007) was that combining 3D electrodes with an appropriate surface functionalization could “trick” neurons or muscle cells to “believe” that the 3D micrometer sized electrodes were neighboring biological entities “that should” be contacted and subsequently actively internalized by endocytotic mechanisms (Spira et al., 2007; Hai et al., 2009a,b).

The specific shape and dimensions of the 3D electrodes was chosen by mimicking the structure and dimension of “spines” that extend from the dendrites of vertebrate neurons (Tonnesen and Nagerl, 2016). Since the 3D electrodes we fabricated bear a resemblance to mushrooms (Figure 1C) and because the term spine-shaped was not familiar to the non-biology members of the MEA community, we referred in subsequent studies to the 3D electrode as gold mushroom-shaped microelectrodes-gMμEs. In addition, to facilitate the adhesion of the cells to the electrode surface and facilitate the gMμE engulfment we covalently functionalized the gMμE by a cysteine terminated arginine-glycine-aspartic acid (RGD) repeat peptide (Spira et al., 2007).

We tested the hypothesis that neurons, “un-professional phagocytes,” can actively engulf micrometer size gMμEs and thereby significantly increase the physical contact between the electrode surface and the cell membrane in two steps. First, because of economic considerations of time and cost, we examined whether cultured *Aplysia* neurons and rat hippocampal cells could endocytose functionalized micrometer size latex beads. These tests proved positive (Figure 1D) and also supported the hypothesis that the chemical functionalization of the beads enhanced the phagocytic activity. Follow-ups by transmission electron microscopic examination of the ultrastructural relationships formed between different cultured cell types and dense arrays of gMμEs (Figure 1E) demonstrated that cultured neurons, primary cardiomyocytes, striated muscle fibers and non-excitable cells (NIH/3T3, CHO, PC-12, H9C2) engulfed gMμEs by forming a reduced cleft width and an increased contact area. Nonetheless, the gMμEs clearly maintained their extracellular position with respect to the cell's plasma membrane. Complementary confocal imaging of *Aplysia* neuron-gMμE hybrids revealed that the formation of the tight physical contact involved the restructuring of the sub-membrane actin skeleton around the gMμE stalk. Quantitative estimates of the seal resistance formed by the neurons and the gMμE suggested that the configuration indeed improved the seal resistance as compared to substrate integrated planar electrodes (Hai et al., 2009a,b, 2010a,b; Ojovan et al., 2015).

## Electrophysiological recordings by gMμE

**Figure 2:**
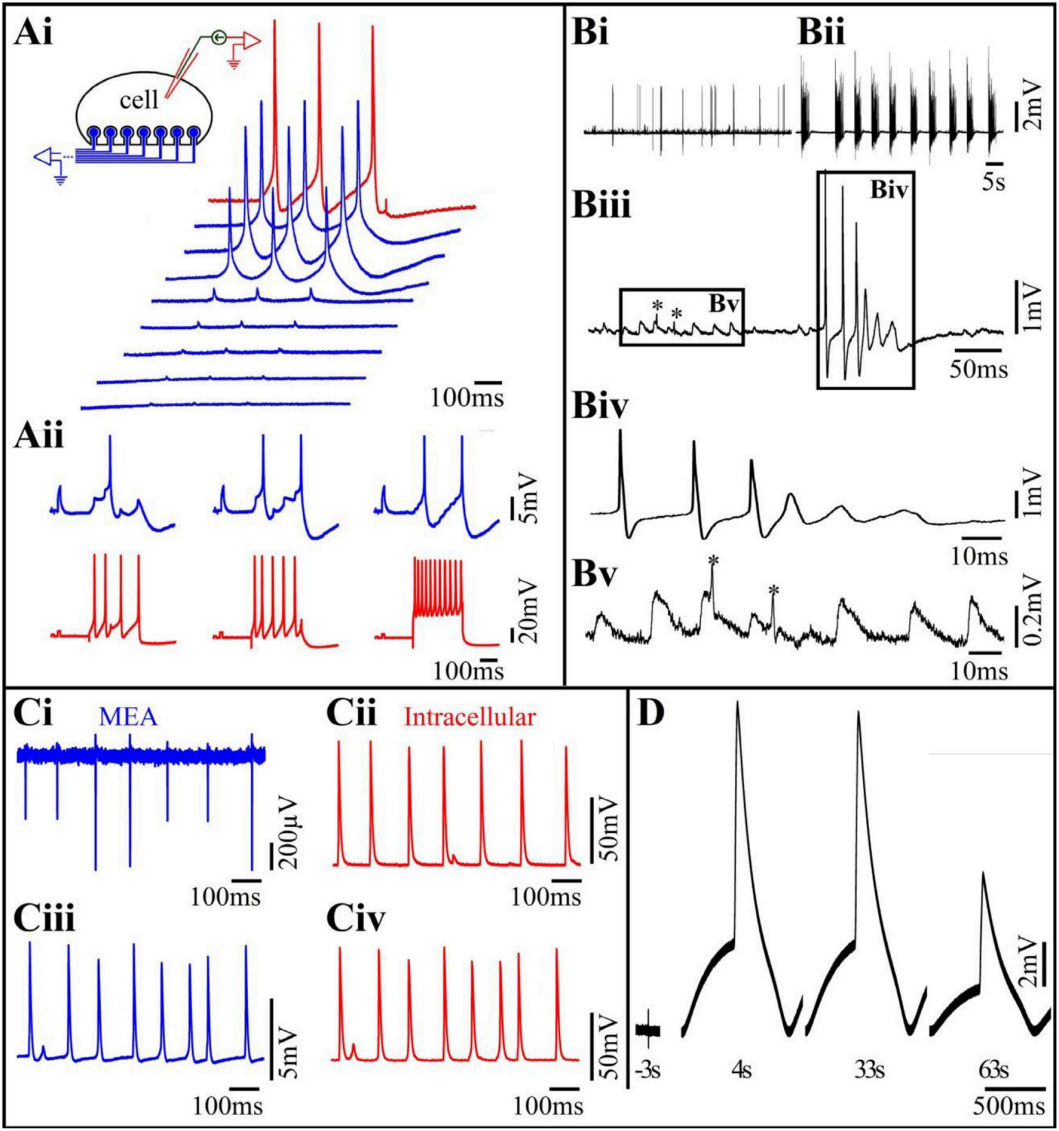
**(Ai)** Differences in the levels of the seal resistance formed between a single Aplysia neuron residing on 8 gMμEs (insert) leads to differences in the IN-CELL recorded APs amplitudes. A cultured Aplysia neuron was intracellularly stimulated to fire 3 consecutive APs (red trace). Simultaneous recordings of these APs by 8 gMμE (blue traces) revealed differences in the IN-CELL recorded amplitudes. **(Aii)** Synaptic- and action-potentials recorded by an extracellular gMμE. Stimulation of a presynaptic neuron by an intracellular sharp electrode (red) lead to the generation of excitatory post synaptic potentials (blue) recorded by a gMμE. Note the summation of the EPSPs to reach firing threshold (**Ai,Aii** modified with permission from Hai et al., 2010a). **(B)** Spontaneous activity recorded by a gMμEs from a cultured hippocampal neuron. **(Bi)** Control spontaneous APs firing recorded by a gMμE, **(Bii)** 10 min after the application of 10 μm GABAzin the firing pattern was changed. **(Biii)** Enlargements of the records in **(Bi)**. **(Biv)** Enlargement of the right box in **(Biii,Bv)** enlargement of the left box in **(Biii)**. The low amplitude long duration potentials in **(Biii)** (left box) have the features of excitatory post synaptic potentials. The fast spikelets indicated by ^*^ could be either dendritic spikes or the firing of electrically coupled neurons. As the dynamics and amplitudes of the potentials shown in **(Bv)** are not altered by GABAzin it is unlikely that they reflect barrage of FPs generated by remote neurons. **(C)** Comparison of intracellular recorded potentials to IN-CELL recordings from cultured myotubes obtained by gMμE electroporation. **(Ci,Cii)** Depict simultaneous extracellular FPs recordings by a gMμE **(Ci)** and intracellular recordings by a sharp electrode **(Cii)**. The recordings **(Ci,Cii)** revealed identical firing patterns and similar qualitative alterations in the amplitudes of the recorded action potentials. **(Ciii)** Electroporation of the myotube changed the recording mode by the gMμE from extracellular to the IN-CELL. Note that although the IN-CELL recorded amplitude of the APs is about an order of magnitude lower than that of the intracellular electrode APs, the shape of the recorded potentials are identical. Also, note that both the gMμE **(Ciii)** and the intracellular sharp electrode **(Civ)** recorded subthreshold potentials in between the APs (**C** modified with permission from Rabieh et al., 2016). **(D)** IN-CELL recordings from cultured human cardiomyocytes. Three seconds before electroporation 4. Thirty-three and sixty-three seconds after electroporation.

A comparison of the electrophysiological recording characteristics obtained by gMμE based-MEA using different cell types provides a better understanding of the strength and limitations of this approach. Using cultured *Aplysia* neurons, we found that within 48–72 h of plating, gMμE functionalized by the RGD-repeat engulfment-promoting-peptide recorded attenuated APs and post synaptic potentials (PSPs) with the characteristic features of intracellular recordings (Figure 2A). Since the gMμE are tightly engulfed by the neurons plasma membrane but remains outside of it, we refer to this mode as “IN-CELL recordings” by extracellular electrodes. This was done to emphasize the way it differs from genuine intracellular recording in which the electrode tip forms direct Ohmic contact with the cytosol. The amplitudes of the IN-CELL recorded APs generated by a single large *Aplysia* neuron (~80 μm in diameter) that contacts a number of gMμEs (spaced 20 μm apart) were not identical and ranged from ~2 to 30 mV. This reflects the variability in the seal resistance formed between the neuron and the various gMμEs (Hai et al., 2010a) and possible differences in the gMμEs' impedances (Figure 2Ai). PSPs of up to 5 mV were recorded from *Aplysia* neurons that were well coupled to the gMμEs as indicated by the high IN-CELL recorded AP amplitude associated with the recorded PSPs (Hai et al., 2010a) and Figure 2Aii). IN-CELL recordings of APs by gMμE that did not undergo chemical functionalization were not generated spontaneously but could be induced to IN-CELL recorded by the delivery of electroporating voltage pulses through the recording gMμE (Hai and Spira, 2012). Using the analog electrical circuit model, we estimated that to account for the experimental results we had to assume that IN-CELL recordings are only possible when in addition to an increased seal resistance, the junctional membrane conductance was increased with respect to the nonjunctional membrane. The mechanism underlying this increase was not investigated but is likely due to the recruitment of voltage independent ionic channels to the junctional membrane or to the formation of nanopores within the confined region of the junctional membrane. Both mechanisms can be triggered by the curvature of the membrane along the gMμE cap and stalk (Petrov et al., 1989; Zhao et al., 2017).

The studies conducted using *Aplysia* neurons revealed that the neuron-gMμE junction was stable for approximately 2 weeks and that the neurons-gMμE hybrid configuration did not alter the passive and active membrane properties of the neurons and their synaptic functions (Hai et al., 2009b).

The results obtained using primary cultured rat embryonic hippocampal neurons differed in a number of ways from those of *Aplysia* neurons. Unlike *Aplysia* neurons that are isolated from mature nerve systems and thus fire full-blown APs at the time of culturing and establish functional synaptic contacts within 12–24 h after plating, neurons derived from embryonic rats are not fully differentiated and require 10–14 days in culture to differentiate morphologically, express excitable membrane properties and form functional synapses. Contrasting with cultured *Aplysia* neuron cultures, the variability in the shapes and amplitude of the IN-CELL recorded potentials from cultured hippocampal neurons was significantly larger, and ranged from biphasic extracellular field potentials with amplitudes in the range of 100 μV (very much like those recorded by substrate integrated planar electrodes), to positive monophasic IN-CELL recorded APs of up to 5 mV (Shmoel et al., 2016, and Figure 2B here). The large variability in the recording mode (extracellular to IN-CELL) from hippocampal neurons can be attributed to two factors. Because of the relatively small diameter of hippocampal neuron cell-bodies, the probability of a given neuron to be optimally positioned with respect to a gMμE (spaced at 100 μm) and fully engulf it is lower than for the large diameter *Aplysia* neurons (Ojovan et al., 2015). Thus, the probability for hippocampal neurons to generate a sufficiently large seal resistance is smaller than for *Aplysia* neurons. In addition, unlike in *Aplysia* neurons even in cases in which a high seal resistance was formed between a neuron and the gMμEs, as indicated by the amplitude of the recorded potential, the resistance of the membrane patch facing the gMμE (R_jn_) could remain high. Under these conditions the electrical coupling between the neuron and the gMμE remained (partially or fully) capacitive rather than Ohmic and the AP shape appeared to be similar to the time derivative of an intracellular AP (Figure 4 in Fromherz, 2003; Shmoel et al., 2016).

Whereas in cultured *Aplysia* neurons IN-CELL recorded post synaptic potentials can be identified by their shape, amplitude and temporal relationships with evoked presynaptic APs, the unequivocal identification of IN-CELL recorded PSPs from hippocampal neurons is by far more complex. The complication stems from the fact that the shape of FPs generated by spontaneous bursts that are remote from the recording site is not easily distinguishable from barrages of PSPs (Shmoel et al., 2016 and Figure 2B here). Nevertheless, as outlined in the legend to Figure 2B, and quantitatively estimated (Ojovan et al., 2015), PSPs can be recorded by extracellular gMμEs when the neuron- gMμEs coupling coefficient is high and the J_mem_ conductance is sufficiently low.

Interestingly, contrary to what we found in *Aplysia* the electrical coupling levels and mode of recording formed between hippocampal neurons and gMμEs were not improved by functionalizing the gMμE with the RGD repeat peptide. Ultrastructural observations by our lab and others have revealed that hippocampal neurons (and cardiomyocytes) engulf and tightly interface gMμEs functionalized by poly-L-lysine or poly-ethylene-imine/laminin, suggesting that the 3D structure in itself (mushroom-, or a nanopillar-shaped protrusion) is sufficient to trigger the engulfment (Santoro et al., 2013, 2014; Zhao et al., 2017). It is conceivable that the expected effects of the RGD repeat peptide on the junctional membrane conductance of hippocampal neurons is not expressed by the time the hippocampal network matures its electrophysiological functions (10–15 days after plating). The peptide layers at the gold electrode surface could be degraded by enzymes secreted by the cells or by hydrolysis. Attempts to achieve Ohmic contact between gMμEs and the neurons by electroporation were unsuccessful. We observed that hippocampal neurons- gMμE hybrids can be stable for periods of up to 10 days but this issue requires further examination. We have not yet tested whether gMμEs alter the physiological properties of the neurons or the network.

Studies on the use of gMμE arrays to record from contracting cardiomyocytes and striated muscle fibers (Figures 2C,D) revealed that both cell types engulf gMμEs functionalized by laminin, this making it possible to record high signal to noise ratio, extracellular FPs generated by individual cells for a number of days (Figure 2Ci). In a fraction of the experiments, striated muscles spontaneously formed IN-CELL recording configurations that allowed recordings of APs with amplitudes of 5–10 mV (Rabieh et al., 2016). In both muscle cell types, a short electroporation pulse delivered through a gMμE converted extracellular recordings to IN-CELL recordings (Figures 2Cii,D). The shape and duration of the recorded IN-CELL APs were similar to those recorded intracellularly (Figure 2C). Interestingly as in the cases of IN-CELL recordings generated by electroporation of cultured *Aplysia* neurons, the increased junctional membrane conductance of the electroporate cardiomyocytes and striated muscles spontaneously recovered, leading to a reversal of the IN-CELL recording mode to extracellular. Electroporation induced in-cell recordings followed by recovery could be repeated a number of times over a few days.

## Conclusion

A diverse series of experiments demonstrated that the concept of attenuated intracellular recordings by extracellular MEA (IN-CELL recordings) can be applied successfully to various neurons, contracting cardiomyocytes and striated muscles under *in vitro* conditions. Thus, the application of the method should be a “game changer” in terms of enhancing our understanding of the physiological mechanisms underlying *in vitro* excitable cell network computations, and long-term changes such as in learning and memory. Planar MEA platforms suffer from low signal to noise ratio and low source resolution (Seymour et al., 2017). These drawbacks are solved by tedious spike-detecting, spike-sorting and signal averaging techniques which rely on estimated parameters. The IN-CELL technique may help circumvent this hurdle.

The recent development of biotechnologies that use induced human pluripotent stem cells taken from healthy subjects and patients, and *in vitro* drug screening for the development of personalized medicine will benefit as well since the IN-CELL recording method makes it possible to extract significantly more information with respect to planar MEA.

There are three focused methodological issues that should nevertheless be addressed before the *in vitro* IN-CELL approach can be used in practice. These are: (a) the development of reliable methods to accurately position and maintain the cultured cells in close proximity to the target gMμE, (b) the development of methods to locally increase the junctional membrane conductance without inducing cell-repair mechanisms that recover the j_mem_ conductance, and (c), the reduction of gMμE impedance.

## Author contributions

All authors listed contributed to the writing of the manuscript and approved it for publication.

### Conflict of interest statement

The authors declare that the research was conducted in the absence of any commercial or financial relationships that could be construed as a potential conflict of interest.
